# Supplementary material for: The pattern of orthopedic fractures and visceral injury in road traffic crash victims, Addis Ababa, Ethiopia
Source: PLoS One. 2021 Sep 24;16(9):e0253690. doi: 10.1371/journal.pone.0253690 (PMC8462740; doi:10.1371/journal.pone.0253690)
Supplement: S1 Dataset — (DOCX) [file pone.0253690.s001.docx]

**Appendex I: Questionnaire form for road traffic crash orthopedic injury survey**

Questionnaire form for road traffic crash involved individual

Case number ______ Date of interview (dd/mm/yy) ______/______/________

| Question **:**  **Section one** – demographic data | Response |
| --- | --- |
| 1. What old are you? | ______(in years) |
| 1. What is your sex? | 1/ male 2/ female |
| 1. Highest level of formal education attained | 1/Illiterate 2/Primary school 3/Secondary school 4/Diploma 5/degree 6/Master 7/Other(specify) _ |
| 1. What is your religion? | 1/ Orthodox 2/Muslim  3/ Protestant 4/ others specify____ |
| 1. What is your marital status? | 1/ single 2/ Married  3/ separated/divorced 4/windowed |
| **Section two:** Road Crash Related data   1. Who was the mobile person in crash involved trip? | 1/Pedestrian 2/Driver 3/ Passenger 4/Other: specify______ |
| 1. Did you have a seatbelt on at time of crash? | 1/ Yes 2/ No 3/ others _______ |
| 1. How did the crash/collision happen (the mechanism of injury)? | 1.car to car collisions 2/ Run off Road  3/ Collision with road side objects 4// Rollover  5/ejection 6/Collision with pedestrian  7/other ___ |

| **Section three**: Injury Related Factors   1. Site of injuries among the victims | Site | Injury severity |
| --- | --- | --- |
|  | Head-neck  Face  Chest  Abdomen  pelvis  Extremity (upper/lower) | ____  ___  ____  ______ |
| 1. Diagnose at admission | _____ | |
| 1. Final diagnose | __ | |
| 1. Diagnosed Isolated fracture | _______________ | |
| 1. Type of fracture | 1/ simple  2/ compound | |
| 1. Number of fractures | 1. Single bone 2. Two bone 3. multiple bone | |
| 1. Diagnosed dislocation | _________ | |
| 1. Diagnosed Visceral injury | ______________ | |
| 1. Discharge status of patient | 1. Well 2. death __ Time/date of death______ 3. other ________ | |
| 1. If death   Specify location of death | 1. Found dead 2/ Died at scene  3/Dead on arrival at hospital  4/ Died in hospital | |
