# Supplementary material for: The pattern of orthopedic fractures and visceral injury in road traffic crash victims, Addis Ababa, Ethiopia
Source: PLoS One. 2021 Sep 24;16(9):e0253690. doi: 10.1371/journal.pone.0253690 (PMC8462740; doi:10.1371/journal.pone.0253690)
Supplement: Attachment — Submitted filename: Response to Reviewers.docx [file pone.0253690.s002.docx]

**Response to Reviewers**

**Subject**: Response to reviewer’s comments

**Manuscript Number**: PONE-D-20-25631-[EMID: b048671f7654c8c2]

**Manuscript Title:** **Pattern of Orthopedic Fractures and Visceral Injury in Road Traffic Crash Victims in Addis Ababa City Administration, Ethiopia.**

Dear: Reviewers

PLOS ONE

Thank you very much for your efforts in handling our manuscript. We hereby acknowledge the positive comments and considerations received from reviewers and editor to improve the contents of our manuscript. We gratefully think that the comments have helped us to further enhance and strengthen the paper. The required amendments have been performed and the revised sections were highlighted throughout the manuscript. We hope that the revised manuscript is now acceptable for publication in your journal. Please find below our response/rebuttal to the reviewers’ comments.

The revised manuscript is submitted for your positive consideration and acceptance.

Sincerely

Zuriyash Mengistu

|  | **REVIEWER COMMENTS TO THE AUTHORS:** | **Response** |
| --- | --- | --- |
| 1 | **Reviewer #1:**  However, in the paper, more works on analysis of traffic injury severity-based logit/probit models should be acknowledged. | **Response to Reviewer #1:**  Ordered probit medel is used  for crash injury severity |
| 2 | **Reviewer #2:**  1. The figures and tables in the manuscript are not standardized and unified, and the list of references should be consistent with the requirements of the journal.  2. The authors did not pay attention to the grammatical mistakes and conciseness of the paper, there are many sentences that are too long and wordy. It is suggested that the author should read it carefully and repair it.  3. Please add two indicators of reliability and validity to test the design quality of the questionnaire  4. Page 26, “Several factors were significant in explaining which factors are more associated with visceral injury using multilevel analysis”. What are the specific factors? In the discussion section, It is suggested that the author should add list and explain which factors are related to visceral injury instead of a brief statement.  5. Also on page 26, “The collusion intensity might be decreased due to advanced age’s calmness to control the vehicle speed, traffic negotiation, risky behavior and impatience”. Please add more reasonable explanation or evidence from previous studies to confirm this result.  6. | **Response to Reviewer #2:**   1. **Revised** 2. Revised   3. The content of questionnaire were extracted from relevant literature review and pretested. The injury severity score criteria: mild injury and above were established. Correlation analysis was done to eliminate correlated explanatory variable such as passenger and vehicle collusion without side object, the significant factor passenger retained in injury severity study  4. specific factors i.e. male sex, age 18 -29 years, compound type of fracture, higher educational status and the group effect ( road sleep or sitting type of road user) were associated factor that were listed and discussed.  5. evidence from previous study was added  6. yes |
