# Supplementary material for: The pattern of orthopedic fractures and visceral injury in road traffic crash victims, Addis Ababa, Ethiopia
Source: PLoS One. 2021 Sep 24;16(9):e0253690. doi: 10.1371/journal.pone.0253690 (PMC8462740; doi:10.1371/journal.pone.0253690)
Supplement: Attachment — Submitted filename: Response to Reviewers.docx [file pone.0253690.s003.docx]

**Response to Reviewers**

**Subject**: Response to reviewer’s comments

**Manuscript Number**: PONE-D-20-25631R1

**Manuscript Title:** The **Pattern of Orthopedic Fractures and Visceral Injury in Road Traffic Crash Victims in Addis Ababa City Administration, Ethiopia.**

Dear: Reviewers

PlOS

|  | **REVIEWER COMMENTS TO THE AUTHORS:** | **Response** |
| --- | --- | --- |
|  | **Reviewer #1:**   1. Manuscript presented in an intelligible fashion and written in standard English 2. The specification of the ordered Logit model is vague. There are some errors in the formulations. 3. Uploading the figure files to the Preflight Analysis and Conversion Engine (PACE) digital diagnostic | **Response to Reviewer #1:**   1. Revised 2. The ordered probit model is was revised. 3. Done |
|  |  |  |
