# Supplementary material for: The pattern of orthopedic fractures and visceral injury in road traffic crash victims, Addis Ababa, Ethiopia
Source: PLoS One. 2021 Sep 24;16(9):e0253690. doi: 10.1371/journal.pone.0253690 (PMC8462740; doi:10.1371/journal.pone.0253690)
Supplement: Attachment — Submitted filename: Response to Reviewers.docx [file pone.0253690.s004.docx]

**Response to Reviewers**

**Subject**: Response to reviewer’s comments

**Manuscript Number**: PONE-D-20-25631R1

**Manuscript Title:** The **Pattern of Orthopedic Fractures and Visceral Injury in Road Traffic Crash Victims in Addis Ababa City Administration, Ethiopia.**

Dear: Reviewers

PlOS

|  | **REVIEWER COMMENTS TO THE AUTHORS:** | **Response** |
| --- | --- | --- |
|  | Journal Requirements:   - Reference review   **Reviewer #1:**  The specification of the ordered logit model  How is the latent variable Y_*_i linked the observed crash injury severity? | Response to journal  References revised, ref 6 and ref 25 removed and replaced  **Response to Reviewer #1:**  The specification of the ordered logit model  revised |
|  |  |  |
